# Studies on Polyethers Produced by Red Algae

**DOI:** 10.3390/md8041178

**Published:** 2010-04-07

**Authors:** Francisco Cen-Pacheco, Laurette Nordström, María Luisa Souto, Manuel Norte Martín, José Javier Fernández, Antonio Hernández Daranas

**Affiliations:** 1 Instituto Universitario de Bio-Orgánica “Antonio González” (IUBO), Universidad de La Laguna (ULL), Astrofísico Francisco Sánchez 2, 38206 La Laguna, Tenerife, Spain; 2 Departamento de Ingeniería Química y Tecnología Farmacéutica, Universidad de La Laguna (ULL), Astrofísico Francisco Sánchez 1, 38071, La Laguna, Tenerife, Spain

**Keywords:** polyether, marine compound, triterpene, Laurencia viridis

## Abstract

Two novel squalene-derived triterpenes, spirodehydrovenustatriol (**3**) and 14-keto-dehydrothyrsiferol (**4**) were isolated from the red alga *Laurencia viridis*, together with two new and unusual C_17_ terpenoids, adejen A (**5**) and B (**6**). These truncated structures possess structural similarities with other known squalene metabolites and their biogenetic origin has been proposed on the basis of an oxidative process of the squalene skeleton. All the structures were elucidated by extensive use of 2D NMR spectroscopic methods.

## 1. Introduction

The Canary Current is characterised by an intense meso-scale structure in the transition zone between the cool and nutrient-rich water of the coastal upwelling regime and the warmer oligotrophic water of the open ocean. The Canary Islands, which straddle the transition, introduce a second source of variability by perturbing the general southwestward flow of both ocean current and trend winds. The combined effects of the flow disturbance and the eddying and meandering of the boundary between upwelled and oceanic waters produce a complex pattern of regional variability [1].

**Figure 1:**
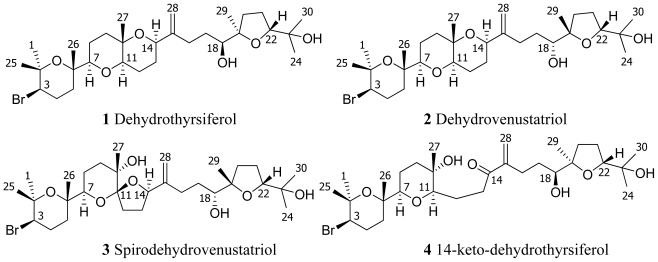
Representative polyether metabolites and new triterpene compounds isolated from *Laurencia viridis*.

From this very brief description of the Canary’s coastal environment, it is easy to understand that finding a large variety of microecosystem with a rich marine biodiversity, is possible. Within these indigenous species, *Laurencia viridis* is a seasonal alga that grows on basaltic rocks in the lower intertidal zone during early spring when the costal temperature is about 18 °C, moderate wind forces and the resulting convection have a maximum penetration into the surface mixed layer [2]. From this alga, we have isolated a complex series of polyether secondary metabolites derived from squalene. These metabolites show a large diversity of ring sizes and functionalization [3–10]. Dehydrothyrsiferol (**1**) and dehydrovenustratriol (**2**) differentiated only by the configuration of carbons C-18 and C-19 are probably the best-known metabolites of these series (Figure 1). Furthermore, important pharmacologic properties as potent cytotoxic effects, protein phosphatase type 2A inhibition and integrin antagonist activity have been described for them [11–14].

In addition to previous work, we now report the isolation of four new compounds from this algae: spirodehydrovenustatriol (**3**) and 14-keto-dehydrothyrsiferol (**4**) belonging to the venustatriol and thyrsiferol series, respectively; in addition to two unusual the C_17_ terpenoids, adejen A (**5**) and B (**6**). These structures were fully established from their spectral data, and the relative stereochemistries and 3D structures were proposed on the basis of ROESY, NOEDIFF analysis and conformational search studies.

## 2. Results and Discussion

Spirodehydrovenustatriol (**3**) was isolated as an amorphous white solid, [α]^25^_D_ + 4.3 (*c* 0.61, CHCl_3_) and its molecular formula was established as C_30_H_51_O_7_Br by ESI-HRMS. The MS data were further supported by the analysis of the ^13^C-NMR chemical shifts of **3**, where seven methyl, eleven methylene, and five methine groups, as well as six oxygenated and one olefinic quaternary carbons were identified (Table 1).

**Figure 2:**
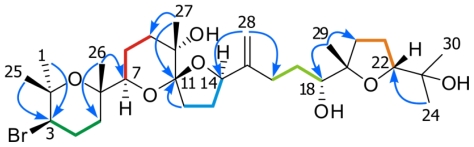
Structure of spirodehydrovenustatriol (**3**). ^1^H-^1^H spin systems are represented by coloured bold lines, while important HMBC correlations are represented by arrows.

Comparison of the NMR spectral data of compound **3** with those reported for dehydrovenustatriol (**2**) and derivatives isolated in our laboratory [5–10], showed some differences around the C-7→C-14 fragment. On the other hand, fragments C-1→C-6 and C-15→C-24 turned out to be identical to those observed in dehydrovenustatriol (**2**) [9]. Analysis of the COSY spectrum allowed us to determine the connectivity’s within the five ^1^H-^1^H spin systems present in this molecule, giving the partial structures shown in Figure 2. The proton assignments in the C-7→C-14 region may be conveniently started from H-7 (Δ_H_ 3.65, d, *J* = 7.0 Hz), which was coupled with H_2_-8 (Δ_H_ 1.78/1.87), and these sequentially to H_2_-9 (Δ_H_ 1.59/1.88). Within the next spin system, H_2_-12 (Δ_H_ 1.87/2.09) was coupled to H_2_-13 (Δ_H_ 1.78/2.07), and these in turn to H-14 (Δ_H_ 3.98, dd, *J =* 5.0, 8.4 Hz). The HMBC correlations of the protons H_2_-9 and H_3_-27 (Δ_H_ 1.11) with the quaternary carbon C-10 (Δ_C_ 72.9) as well as those the protons H_2_-12, H_2_-13 and H_3_-27 with C-11 (Δ_C_ 109.9), positioned the methyl group C-27 at the oxygen-bearing carbon C-10 and linked both partial structures through the ketal quaternary carbon C-11, indicating at the same time that both rings are linked by a spiroketal at this position.

**Figure 3:**
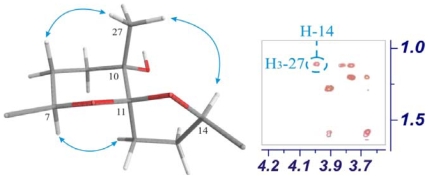
Important ROESY correlations observed for B-C ring system in compound **3**.

The relative configuration of the stereocentres C-3, C-6, C-7, C-10, C-18, C-19 and C-22 were established as identical to those found in venustatriol series on the basis of correlations observed in the ROESY experiment as well as through interpretation of NMR coupling constants data [5,9]. Furthermore, the relative configuration of the new spiroketal carbon was established as S* on the basis of the cross-correlation peak observed in the ROESY experiment between protons, H-14 and H_3_-27 that can only be explained by the proposed orientation as is shown in Figure 3.

**Figure 4:**
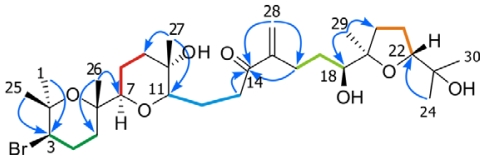
Structure of 14-keto-dehydrothyrsiferol (**4**). ^1^H-^1^H spin systems are represented by coloured bold lines, while important HMBC correlations are represented by arrows.

The next compound, 14-keto-dehydrothyrsiferol (**4**), proved to have the same molecular formula as **3**, (C_30_H_51_O_7_Br) on the basis of the result obtained from ESI-HRMS. Nevertheless, comparison of the ^13^C-NMR spectral data between compounds **3** and **4** showed the absence of the characteristic spiroketal carbon C-11 (Δ_C_ 109.9) as well as the existence of a new carbonyl signal at Δ_C_ 202.3 together with the absorption in the UV spectrum characteristic of an α,β-unsaturated ketone. These data, in concert with the interpretation of the 2D NMR spectra and comparison with the previously reported compounds [5–10], clearly indicated that the main differences between both compounds where located at the C-7→C-14 fragment (Table 1). Thus, the connectivity’s observed in the COSY and HSQC experiments made it possible to assign these fragments as follows: the first spin system was started by the methine proton H-7 (Δ_H_ 2.97, dd, *J =* 1.7, 11.0 Hz) coupled with both H_2_-8 (Δ_H_ 1.38/1.76), which were in turn correlated to H_2_-9 (Δ_H_ 1.48/1.82). The next spin system was formed by the methine proton H-11 (Δ_H_ 3.03, dd, *J =* 1.2, 10.3 Hz) that connected with H_2_-12 (Δ_H_ 1.51/2.01). These were further correlated to H_2_-13 (Δ_H_ 2.71/2.87) (Figure 4). These substructures were joined together using the HMBC experiment whereby the protons H_3_-26 (Δ_H_ 1.19) were correlated with carbons C-5 (Δ_C_ 36.7), C-6 (Δ_C_ 74.6) and C-7 (Δ_C_ 86.1); the signal of H_3_-27 (Δ_H_ 1.15) showed correlations with C-9 (Δ_C_ 39.8), C-10 (Δ_C_ 69.9) and C-11 (Δ_C_ 83.7); and the typical carbonyl signal centred at Δ_C_ 202.3 (C-14) was correlated from the protons H_2_-13, H_2_-16 (Δ_H_ 2.39/2.54) and H_2_-28 (Δ_H_ 5.80/6.01). Finally, analysis of the ROESY experiment confirmed the relative stereochemistry of all chiral centres presented in the molecule as equivalent to those observed in the metabolites belonging to the thyrsiferol series. It has to be noted that this metabolite has a special interest from a biogenetic point of view, with regard to the next two compounds described in this paper.

The molecular formulae of adejen A (**5**) and B (**6**), C_17_H_27_O_3_Br and C_17_H_27_O_4_Br, respectively, together with the fact that their ^1^H- and ^13^C-NMR spectra were reminiscent of the corresponding partial spectral signals of thyrsenol A led us to a quick identification of the structures of both compounds [7]. Both compounds share identical A-B ring moiety, whereas the only notable differences were fixed going towards the methine group at carbon C-11. In adejen A (**5**), the COSY spectrum revealed coupling between the methine proton H-11 (Δ_H_ 3.35, dd, *J =* 5.8, 10.6 Hz) and the allylic methylene signals H_2_-12 at Δ_H_ 1.88/2.04, which were in turn coupled to the olefinic proton H-13 (Δ_H_ 4.58, ddd, *J* = 2.0, 5.8 and 5.8 Hz) implicated in a *Z* olefin together with H-14 (Δ_H_ 6.14, ddd, *J* = 1.4, 2.6 and 5.8 Hz) (Table 2). Furthermore, the characteristic chemical shifts of C-13 and C-14 at Δ_C_ 98.1 and 141.7 respectively; in addition with the HMBC correlations for the bearing oxygen C-10 (Δ_C_ 74.3) with the olefinic proton H-14 indicated that this compound includes an enol-ether ring system.

A similar analysis for adejen B (**6**) revealed that H-11 centred at Δ_H_ 3.43 (dd, *J =* 5.4, 12.1 Hz) was connected with protons H_2_-12 at Δ_H_ 1.80/1.90 using the COSY experiment, that are also coupled with the methylene protons H_2_-13, Δ_H_ 2.66/2.75. In addition, H_2_-13 showed correlations in the HMBC with a carbonyl signal at Δ_C_ 170.2 (C-14), a clue that, in agreement to the strong band observed in the IR spectra at 1739 cm^−1^, established the existence of a lactone moiety. The proposed structures for compounds **5** and **6**, including the same relative configuration found either in the thyrsiferol or venustatriol series, were consistent with the correlations observed in the ROESY experiment.

From a biogenetic point of view, the discovery of the compounds described here support our previous proposal that the cyclization mechanism should be sequential as opposed to the classic hypothesis involving a concerted biogenetic mechanism. The discovery of a biogenetic intermediate such as **4** with a carbonyl group at C-11 should be considered the key to explain the formation of the compound such as thyrsenol A [6]. The compounds that possess an unusual truncated C_17_ carbon skeleton may be their biogenetical origin is 14-keto-dehydrothyrsiferol (**4**), that may arise from a oxidative degradation followed by a cyclization process to become the corresponding enol-ether **5** or lactone ring **6**, respectively.

**Figure 6:**
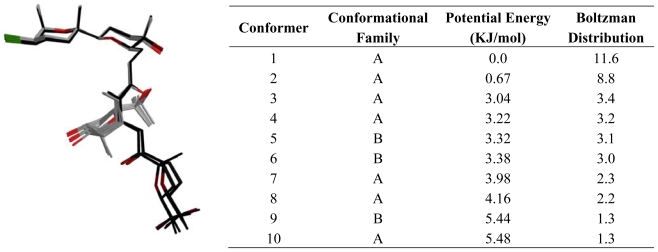
Conformational analysis results of 14-keto-dehydrothyrsiferol **(4**). The two low energy conformation of 14-keto-dehydrothyrsiferol in CDCl_3_ solution. Conformational Family: **A** (C: gray, O: red, Br: green); **B** (C: black, O: garnet, Br: green).

**Figure 7:**
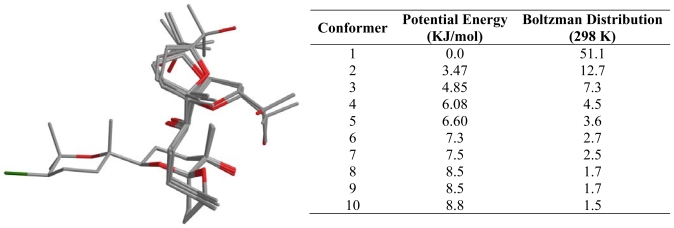
Conformational analysis results of spirodehydrovenustatriol (**3**) in CDCl_3_ solution. (C: gray; O: red; Br: green).

**Figure 8:**
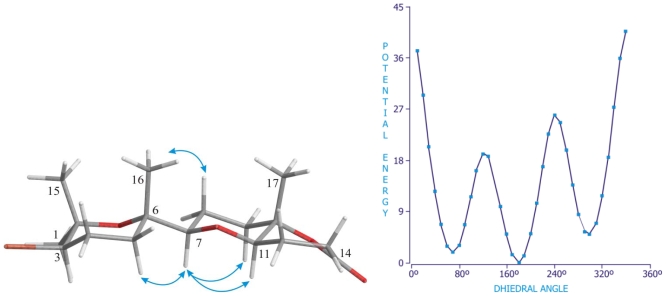
Best energy structure found for C_17_ terpenoid, adejen B (**6**). Significant ROE correlations are shown in the Newman projection of the C-6**–**C-7 bond.

Once the planar structure and the relative stereochemistry of compounds **3** to **6** were determined, a conformational study was carried out in order to corroborate our previous hypothesis about the importance of the orientation of the C-15 to C-25 flexible moiety in the cytotoxic activity of these polyethers [11]. Thus, the crystal structure of 23-thyrsiferyl acetate was used as a template to build the structures by removal of the appropriate covalent bonds [15]. The chirality of the new stereocentres was then adapted according to the experimental data and the resulting structures were used as the starting point for the conformational searches (Figures 6–8).

Based on previous results obtained in our laboratory with this kind of molecules, two independent conformational searches for compounds **3** and **4** using the MMFF94s [16] force field as implemented in MacroModel 8.5 using the generalized Born/surface area (GBSA) solvent model for chloroform were undertaken [11,16]. Random searches of 10,000 MCMM steps were undertaken for each compound to ensure that the potential energy surface was explored using the TNCG algorithm. All local minima within 50 kJ of the global minimum were saved and subsequently re-minimized using the FMNR algorithm and an energy cutoff of 25 kJ to save the resulting molecules. On the other hand, for compounds **5** and **6**, due to their conformational limitations, we used a systematic search around the only single bond connecting C-6 and C-7.

With regard to **4**, our conclusion after an analysis of the conformational search results was that this molecule is likely to exist in a fast conformational equilibrium between two different families of structures as shown in Figure 6. In fact, it can be calculated from the estimated populations by a Boltzmann distribution at 300 K that these conformational families constitute 53.7% (A) and 23.9% (B), of all structures within a 10 kJ/mol cut-off. On the other hand, the results obtained from the conformational search undertook for **3** showed a molecule with a less complicated conformational behavior. Even though **3** has a long acyclic moiety between C14 and C19, it seems that it clearly adopts a preferred “global folding” in solution, where only the C18-C19 bond appears to fluctuate. As a result the C19-C22 ring occupies two different positions.

Finally, for adejen A (**5**) and B (**6**), the result of the systematic conformational search indicated that the global minimum corresponds to a value of ~180° for the dihedral angle C-16**–**C-6**–**C-7**–**H-7 as showed in Figure 8. The obtained results are in full agreement with the ROESY derived data for both compounds, giving confidence in the theoretically obtained structures.

Biological assays of the pure compounds 3, 5 and 6 were undertaken. Cytotoxic effects were evaluated with a couple of breast cancer cell lines (Hs578T and T47D) due to the well-known activity of this kind of compounds against them [5]. However, the result of these bioassays was that none of the studied compounds showed any activity below the 10 μg/mL concentration limit.

## 3. Experimental Section

### 3.1. General methods

Optical rotations were determined on a Perkin-Elmer 241 polarimeter. IR spectra were measured on a Bruker IFS55 spectrometer. The NMR spectra were obtained with a Bruker 500 AMX, and Bruker 400 and 300 Advance instruments. Chemical shifts are reported relative to TMS and coupling constants are given in Hz. HRMS were performed on a VG AutoSpec FISON spectrometer. HPLC was carried out with a LKB 2248 system equipped with a differential diffractometer detector. Silica gel CC and TLC were performed on Silica gel Merck 60 G. TLC plates were visualised by spraying with H_2_SO_4_/H_2_O/AcOH (1:4:20) and heating.

### 3.2. Plant material

The specimens of Laurencia viridis were collected in March 2008 in Callao Salvaje, Paraiso Floral, Adeje (Tenerife, Canary Island). A voucher specimen was deposited at the herbarium of the La Laguna University, Department of Vegetal Biology, Botany, Tenerife).

### 3.3. Extraction and chromatographic separation

The fresh alga was extracted with a 1:1 mixture of CHCl_3_-MeOH at room temperature. The extract was concentrated to yield a crude extract of 83.0 g. This material was chromatographed on a Sephadex LH-20 column using CHCl_3_-MeOH (1:1). Fractions that were similar in composition as shown by TLC were combined to give four fractions. The second fraction (53.4 g) was further separated by silica gel eluted with increasing concentrations of EtOAc in *n*-hexane followed by a medium pressure chromatography Lobar LiChroprep-RP18 with H_2_O-MeOH (9:1) as eluent. Final purification was carried out by HPLC employing μ-Porasil column and using n-hexane-EtOAc in different proportions affording the pure new compounds spirodehydrovenustatriol (**3**, 6.1 mg), 14-ketodehydrothyrsiferol (**4**, 4 mg), **5** (3.0 mg) and **6** (2.6 mg).

#### Spirodehydrovenustatriol (**3**)

Amorphous white solid; [α]^25^_D_ + 4.3 (*c* 0.61, CHCl_3_); IR*ν*_max_ (CHCl_3_) 2928, 2858, 1729, 1590, 1468, 1380 and 1038 cm^−1^; ESI-MS *m/z* 609, 607, 585, 545, 425, 413 and 301; ESI-HRMS *m/z* 607.2618 (Calcd. for C_30_H_49_O_6_^79^BrNa, 607.2610, [M−H_2_O+Na]^+^); ^1^H-NMR (400 MHz, CDCl_3_) see Table 1.

#### 14-keto-dehydrothyrsiferol (**4**)

Amorphous white solid; [α]^25^_D_ + 3.9 (*c* 0.18, CHCl_3_); UV λ_max_ (CHCl_3_) 239.8 (ɛ 1630) (log ɛ = 3.2); IR*ν*_max_ (CHCl_3_) 3438, 2973, 2865, 1587, 1443, 1379 and 1094 cm^−1^; EI-MS *m/z* 627, 625, 609, 607, 413, 301 and 172; ESI-HRMS *m/z* 625.2708 (Calcd. for C_30_H_51_O_7_^79^BrNa, 625.2716, [M + Na]^+^); ^1^H-NMR (400 and 500 MHz, CDCl_3_) see Table 1.

#### Adejen A (**5**)

Amorphous white solid; [α]^25^_D_ + 1.9 (*c* 0.30, CHCl_3_); IR*ν*_max_ (CHCl_3_) 2928, 2858, 1728, 1590, 1450 and 1383 cm^−1^; ESI-HRMS *m/z* 381.1035 (Calcd. for C_17_H_27_O_3_^79^BrNa, 381.1041, [M + Na]^+^); ^1^H-NMR (400 and 500 MHz, CDCl_3_) see Table 2.

#### Adejen B (**6**)

Amorphous white solid; [α]25_D_ + 8.6 (*c* 0.26, CHCl_3_); IR*ν*_max_ (CHCl_3_) 3570, 2951, 2349, 1739, 1584 and 1468 cm ^−1^; ESI-HRMS *m/z* 397.0982 (Calcd. for C_17_H_27_O_4_^79^BrNa, 397.0990, [M + Na]^+^); ^1^H-NMR (400 and 500 MHz, CDCl_3_) see Table 2.

### 3.4. Biological activity

Breast cancer cell lines Hs578T and T47D were cultured in Dulbecco’s Minimum Essential Medium (DMEN) supplement with 10% fetal bovine serum (FBS), 2 mM L-glutamine and 100 U/mL penicillin and streptomycin using standard protocol and seeded in 200 μL wells. After preincubation (37 °C, 5% CO_2_ for 24 h), cells were exposed to graded concentrations of compounds in triplicate (37 °C, 5% CO_2_ for 48 h). For quantitative estimation of cytotoxicity, the colorimetric XTT method was used. Thus, the cells were treated with 50 μL of XTT solution (1 mg/mL in PBS) after removal of the medium and incubated for 3 h. Residual formazan was then separated from the aqueous solution with DMSO (100 μL) and the absorbance was measured using a Bio-Rad at 490 nm. IC_50_ values were estimated by plotting absorbance values against concentrations. Adriamycin and DMSO were used as the positive and negative control in this bioassay [18].

## Figures and Tables

**Figure 5:**
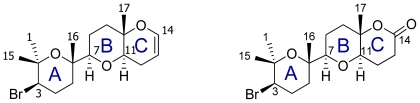
Structure proposed for C_17_ terpenoid compounds **5** (left) and **6** (right).

**Table 1:** NMR chemical shift data (CDCl_3_) for compounds **3** and **4**.

	Spirodehydrovenustatriol (3)				14-Keto-dehydrothyrsiferol (4)			

nº C	δ^13^C	δ^1^H	Mult	*J* (Hz)	δ^13^C	δ^1^H	Mult	*J* (Hz)
1	29.7	1.28 (3H)	s		31.1	1.27 (3H)	s	
2	74.9				75.0			
3	59.1	3.90	dd	4.0;12.1	58.8	3.88	dd	4.0;12.6
4	29.3	2.12(α)/2.23(β)			28.2	2.10(α)/2.25(β)		
5	36.1	1.64 (2H)			36.7	1.55(α)/1.77(β)		
6	74.8				74.6			
7	88.1	3.65	d	7.0	86.1	2.97	dd	1.7;11.0
8	27.6	1.78(β)/1.87(α)			23.5	1.38(β)/1.76(α)		
9	34.3	1.59(α)/1.88(β)			39.8	1.48(α)/1.82(β)		
10	72.9				69.9			
11	109.9				83.7	3.03	dd	1.2;10.3
12	33.7	1.87/2.09			23.8	1.51/2.01		
13	26.6	1.78/2.07			34.6	2.71/2.87		
14	83.3	3.98	dd	5.0;8.4	202.3			
15	145.8				148.5			
16	30.9	2.32/2.57			28.2	2.39/2.54		
17	29.1	1.50/1.82			30.7	1.37/1.60		
18	75.2	3.82	dd	2.5;11.7	75.9	3.48	dd	1.5;10.5
19	84.6				86.0			
20	35.0	1.61/2.01			31.5	1.56/2.09		
21	24.7	1.77 (2H)			26.6	1.83 (2H)		
22	86.7	3.76	dd	7.0;8.3	87.7	3.75	dd	6.1;10.0
23	70.6				70.5			
24	25.4	1.11 (3H)	s		23.9	1.12 (3H)	s	
25	24.1	1.41 (3H)	s		23.5	1.40 (3H)	s	
26	21.1	1.20 (3H)	s		20.3	1.19 (3H)	s	
27	22.7	1.11 (3H)	s		20.1	1.15 (3H)	s	
28	107.0	4.77/4.91	bs/bs		124.5	5.80/6.01	bs/bs	
29	23.9	1.12 (3H)	s		23.9	1.12 (3H)	s	
30	28.2	1.20 (3H)	s		27.7	1.21 (3H)	s	

**Table 2:** NMR chemical shift data (CDCl_3_) for compounds **5** and **6**.

	Adejen A (5)				Adejen B (6)			

nº C	δ^13^C	δ^1^H	Mult	*J* (Hz)	δ^13^C	δ^1^H	Mult	*J* (Hz)
1	31.4	1.27 (3H)	s		30.9	1.27 (3H)	s	
2	75.4				75.1			
3	59.4	3.89	dd	4.1;12.3	58.5	3.88	dd	4.1;12.4
4	28.8	2.11(α)/2.25(β)			28.1	2.11(α)/2.25(β)		
5	37.5	1.53(α)/1.83(β)			36.9	1.54(α)/1.81(β)		
6	74.6				74.1			
7	86.8	3.09	dd	2.5;11.4	86.7	3.15	dd	2.5;11.6
8	23.3	1.51(β)/1.83(α)			22.4	1.47(β)/1.88(α)		
9	36.9	1.61(α)/1.89(β)			36.7	1.74(α)/1.99(β)		
10	74.3				78.7			
11	77.2	3.35	dd	5.8;10.6	76.5	3.43	dd	5.4;12.1
12	24.5	1.88(β)/2.04(α)			21.6	1.80(β)/1.90(α)		
13	98.1	4.58	ddd	2.0;5.8;5.8	28.1	2.66/2.75		
14	141.7	6.14	ddd	1.4;2.6; 5.8	170.2			
15	24.1	1.41 (3H)	s		23.6	1.41 (3H)	s	
16	20.3	1.21 (3H)	s		20.0	1.21 (3H)	s	
17	15.9	1.14 (3H)	s		19.3	1.36 (3H)	s	

## References

[1] Barton ED Aristegui J Tett P Cantón M García-Braun J Hernández-León S Nykjaer L Almeida C Almunia J Ballesteros S Basterretxea G Escánez J García-Weill L Hernández-Guerra A López-Laatzen F Molina R Montero MF Navarro-Pérez E Rodríguez JM Van Lenning K Vélez H Wild K The transition zone of the Canary Current upwelling region Prog Oceanogr 1998 41 455 504

[2] Gil-Rodriguez MC Haroun R *Laurencia viridis* sp. Nov. (Ceramiales, Rhodomelacea) from the Macaronesian Archipielagos Bot Mar 1992 35 227 237

[3] Faulkner DJ Marine natural products Nat Prod Rep 2002 19 1 48 and previous reviews in this series 11902436 10.1039/b009029h

[4] Blunt JW Copp BR Munro MHG Northcote PT Prinsep MR Marine natural products Nat Prod Rep 2010 27 165 237 and previous reviews in this series 20111802 10.1039/b906091j

[5] Fernández JJ Souto ML Norte M Marine polyether triterpenes Nat Prod Rep 2000 17 235 246 10888011 10.1039/a909496b

[6] Norte M Fernández JJ Souto ML García-Grávalos MD Two new antitumoral polyether squalene derivatives Tetrahedron Lett 1996 37 2671 2674

[7] Norte M Fernández JJ Souto ML Gavín JA García-Grávalos MD Thyrsenols A and B, two unusual polyether squalene derivatives Tetrahedron 1997 53 3173 3178

[8] Norte M Fernández JJ Souto ML New polyether squalene derivatives from Laurencia Tetrahedron 1997 53 4649 4654

[9] Manríquez CP Souto ML Gavín JA Norte M Fernández JJ Several new squalene-derived triterpenes from *Laurencia* Tetrahedron 2001 57 3117 3123

[10] Souto ML Manríquez CP Norte M Fernández JJ Novel marine polyethers Tetrahedron 2002 58 8119 8125

[11] Norte M Fernández JJ Souto ML Evaluation of the cytotoxic activity of polyethers isolated from *Laurencia* Bioorg Med Chem 1998 6 2237 2243 9925286 10.1016/s0968-0896(98)80004-7

[12] Souto ML Manríquez CP Norte M Leira F Fernández JJ The inhibitory effects of squalene-derived triterpenes on protein phosphatase PP2A Bioorg Med Chem Lett 2003 13 1261 1264 12657259 10.1016/s0960-894x(03)00136-7

[13] Pec MK Aguirre A Moser-Thier K Fernández JJ Souto ML Dorta JF Díaz-González F Villar J Induction of apoptosis in estrogen dependent and independent breast cancer cells by the marine terpenoiid dehydrothyrsiferol Biochem Pharm 2003 65 1451 1461 12732357 10.1016/s0006-2952(03)00123-0

[14] Pec MK Artwohl M Fernández JJ Souto ML Giradles T Alvarez de La Rosa D Valenzuela-Fernández A Díaz-González F Chemical modulation of VLA integrin affinity in human breast cancer cells Exp Cell Res 2007 313 1121 1134 17331499 10.1016/j.yexcr.2007.01.015

[15] Blunt JW McCombs JD Munro MHG Thomas FN Complete Assignment of the 13C and 1H NMR Spectra of Thursiferyl Acetate Magn Reson Chem 1989 27 792 795

[16] Halgren TA MMFF VI. MMFF94s option for energy minimization studies J Comput Chem 1996 17 490 519 10.1002/(SICI)1096-987X(199905)20:7<720::AID-JCC7>3.0.CO;2-X 34376030

[17] Mohamadi F Richards NGJ Guida WC Liskamp R Lipton M Caufield C Chang G Hendrickson T Still WC Macromodel - an integrated software system for modeling organic and bioorganic molecules using molecular mechanics J Comput Chem 1990 11 440 467

[18] Bergeron RJ Cavanaugh PF Jr Kline SJ Hughes RG Jr Elliott GT Porter CW Antineoplastic and antiherpetic activity of spermidine catecholamide iron chelators Biochem Biophys Res Commun 1984 121 848 854 6331431 10.1016/0006-291x(84)90755-1

